# Alterations in Periostin Expression Associated With Combined Corticosteroid and Antibiotic Therapy in Patients Suffering From Chronic Rhinosinusitis With Nasal Polyps

**DOI:** 10.7759/cureus.108192

**Published:** 2026-05-03

**Authors:** Konstantina Chrysouli, Agapi Kataki, Efthymios Koniaris, Thomas Nikolopoulos, Alexander Delides, Pavlos Maragkoudakis

**Affiliations:** 1 Ear, Nose, and Throat Department, Sismanogleio General Hospital, Athens, GRC; 2 First Department of Propedeutic Surgery, Hippocration Hospital, Athens Medical School, National and Kapodistrian University of Athens, Athens, GRC; 3 Department of Pathology, Hippokration General Hospital, Athens, GRC; 4 Second Ear, Nose, and Throat Department, Attikon University Hospital, National and Kapodistrian University of Athens, Athens, GRC; 5 Second Ear, Nose, and Throat Department, National and Kapodistrian University of Athens, School of Medicine, Attikon University Hospital, Athens, GRC

**Keywords:** bleeding, corticosteroids, functional endoscopic sinus surgery, inflammation, nasal polyps

## Abstract

Introduction: One of the most common complications during endoscopic sinus surgery (ESS) for chronic rhinosinusitis with nasal polyps (CRSwNP) is bleeding, which increases the time of surgery. A strategy for reducing intraoperative bleeding is the administration of preoperative corticosteroids and antibiotics.

Methods: We aimed to assess the degree and intensity of inflammation in CRSwNP histological specimens from patients who received immediate preoperative treatment with oral corticosteroids and antibiotics, compared with those who did not. The study group consisted of 50 patients who received preoperative corticosteroids and antibiotics, and the control group consisted of 50 patients who had not received any pretreatment. Immunohistochemical expression of periostin in nasal polyp tissue from each group was evaluated and compared to assess the prognostic value of this protein.

Results: There is a statistically significant difference between the control group and the study group in periostin expression (increased in the former) (p < 0.001). The pretreatment group has a significantly lower score (study group means H-score = 112.7 (standard deviation (SD) = 40.4)/control group mean H-score = 159.4 (SD = 42.3)). Moreover, a statistically significant positive correlation between periostin concentration and vascularity (microvessel density and optical density of the vascular endothelial marker) in the pretreatment group is not observed (rho = -0.02, p = 0.907; rho = 0.07, p = 0.713, respectively).

Conclusions: The results support the preoperative administration of systemic corticosteroids and antibiotics to reduce the degree and intensity of inflammation in CRSwNP; this may impact the surgical field during ESS. Further cohort studies are needed to validate this recommendation.

## Introduction

Many recent studies have shown that the overexpression of interleukins (ILs) promotes the development and recurrence of chronic rhinosinusitis with nasal polyps (CRSwNP) [1,2]. ILs and corticosteroids are potential targets for new therapies in nasal polyps[3]. Corticosteroids are widely used for the conservative treatment of nasal polyposis. Recently, a preoperative scheme of corticosteroid administration before endoscopic sinus surgery (ESS) has been proposed.

The aim is to study the effect of the premedication with corticosteroids and antibiotics on the degree and intensity of inflammation in CRSwNP. We conducted a retrospective and prospective comparative study of changes in the degree and intensity of inflammation in CRSwNP among patients who received immediate preoperative treatment with oral corticosteroids and antibiotics, compared with patients who did not.

## Materials and methods

Patients and tissue samples

The sample for this study was collected from January 2013 to the present, specifically from the Ear, Nose, and Throat (ENT) Departments of General Hospitals located in the Athens region. A total of 100 patients who underwent surgery after failure of adequate conservative treatment and who, according to international guidelines, needed surgery for a confirmed histological diagnosis of CRSwNP were included.

The study group consisted of 50 patients who received immediate preoperative treatment with per os corticosteroids and antibiotics (methylprednisolone in tapering form and amoxicillin/clavulanic acid) for seven days. More specifically, the first group (study group) was given preoperative preparation with per os corticosteroids (methylprednisolone 16 mg three times a day for three days with gradual reduction (tapering) 16 mg two times a day for the next two days and 16 mg one time a day for the last two days) as well as antibiotics (amoxicillin/clavulanic acid 1 g three times per day) for seven days. The control group consists of 50 patients who have not received any pretreatment.

From the archives of the Pathological Laboratories of the General Hospitals of Athens, the paraffin cubes from the selected patients were retrieved to obtain material for immunostaining. Regarding inflammation, the degree and intensity of immunohistochemical expression of the inflammatory marker periostin in each group were evaluated.

Reagents

The investigation included the detection of periostin with the rabbit polyclonal anti-periostin anti-rabbit antibody from Abcam International Inc. (Cambridge, UK, ab14041) at a dilution of 1:100. EnVision Flex (DM830; Dako, Glostrup, Denmark) was used for dilution. Immunohistochemical staining (IHC) was performed via the peroxidase-labeled streptavidin-biotin technique using the Rabbit-Specific HRP/DAB Detection IHC Kit from Abcam International Inc., Cambridge, UK (AB64261), according to the company's instructions.

Tissue preparation and immunostaining

Paraffin sections were used for histology. Five sections of 5 μm thick were cut from each paraffin cube. The former and the latter were stained with hematoxylin and eosin (H&E) to confirm the presence of representative nasal polyp tissue. In this study, H&E staining was used to evaluate the anti-periostin antibody.

The positivity and intensity of periostin immunoexpression in two groups of patients with CRSwNP were evaluated. The total immunostaining score (positivity and staining intensity) was calculated for each case and derived from the semiquantitative immunohistochemical score (H-score), with values ranging from 0 to 300.

The score was calculated by multiplying the percentage of positively stained cells by the staining intensity using the following formula: $[1 \times (\% \text{ weakly positive area}) {+} 2 \times (\% \text{ moderately positive area}) {+} 3 \times (\% \text{ strongly positive area})]$. Based on the total H-score above, the results were divided into three categories: first <100, second 100-200, and third >200. Subsequently, a comparison between the two groups with respect to periostin immunoexpression was performed.

Statistical analysis

Quantitative variables were expressed as mean values (standard deviation (SD)) and as median (interquantile range). Variables were tested for normality using the Kolmogorov-Smirnov criterion. The Mann-Whitney test was used to compare continuous variables between two groups when the distribution was not normal. All reported p values are two-tailed. Statistical significance was set at p < 0.05, and analyses were conducted using the Statistical Package for the Social Sciences statistical software (version 27.0; IBM Corp., Armonk, NY).

## Results

Demographic data

Distribution by Gender of the Two CRSwNP Groups

In a 100-patient sample, 26% (n = 26) are men and 24% (n = 24) are women without any treatment, while 32% (n = 32) are men and 18% (n = 18) are women with preoperative treatment.

Age Distribution of the Two CRSwNP Groups

The distribution of patients across the two CRSwNP groups by age is shown in Table 1.

**Table 1:** Age distribution of the two CRSwNP groups CRSwNP: chronic rhinosinusitis with nasal polyps

CRSwNP groups	Age group					Total (n = 100)
	<35	36-45	46-55	56-65	>65	
Control group	2	16	13	8	11	50
Study group	5	28	11	4	2	50

Immunohistochemical Findings and Statistical Analysis Results

The sample consisted of 100 patients: 50 who received preoperative preparation with oral corticosteroids (methylprednisolone 16 mg three times per day for three days, followed by a gradual reduction to 16 mg twice per day for the next two days, and 16 mg once per day for the last two days), as well as antibiotics (amoxicillin/clavulanic acid 1 g three times per day) for seven days, while the remaining 50 patients received no treatment. Τotal immunostaining scores of periostin, based on H-score, were compared between the two groups.

Moreover, the possible positive association between periostin concentration and vascularity (microvascular density (MVD) and optical density of the vascular endothelial marker) in CRSwNP with pretreatment was analyzed.

Periostin

Sixty percent (n = 30) of CRSwNP with preoperative corticosteroid administration had a total immunostaining score of 100-200, while 22% (n = 11) of CRSwNP without preoperative corticosteroid administration had a total immunostaining score >200 (Tables 2, 3).

**Table 2:** Periostin-score distribution in CRSwNP with preoperative corticosteroid CRSwNP: chronic rhinosinusitis with nasal polyps

CRSwNP study group (n)	H-score		
	<100	100-200	>200
50	20	30	0

**Table 3:** Periostin-score distribution in CRSwNP without preoperative corticosteroid CRSwNP: chronic rhinosinusitis with nasal polyps

CRSwNP control group (n)	H-score		
	<100	100-200	>200
50	3	36	11

**Figure 1:**
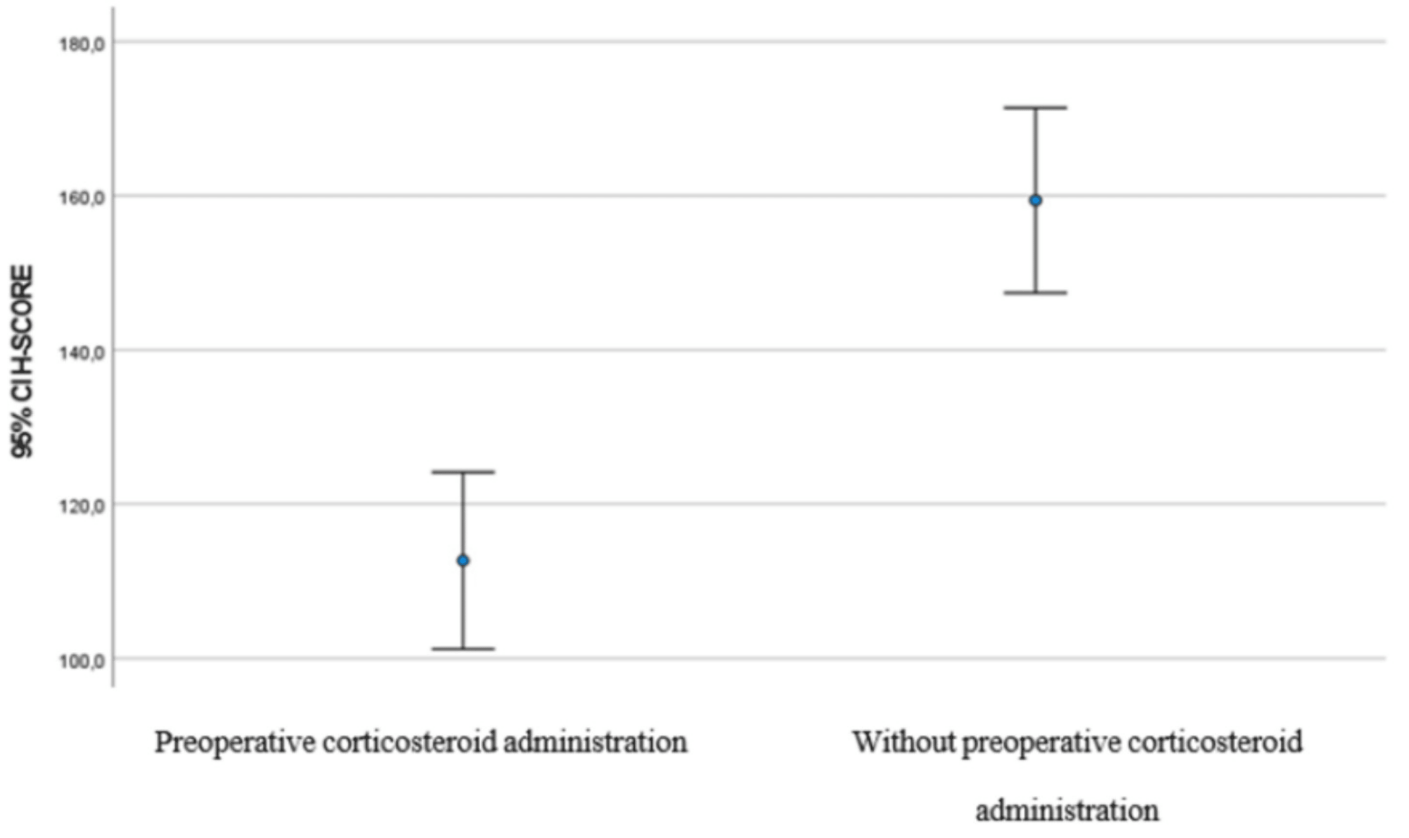
Error bar for H-score by group

**Figure 2:**
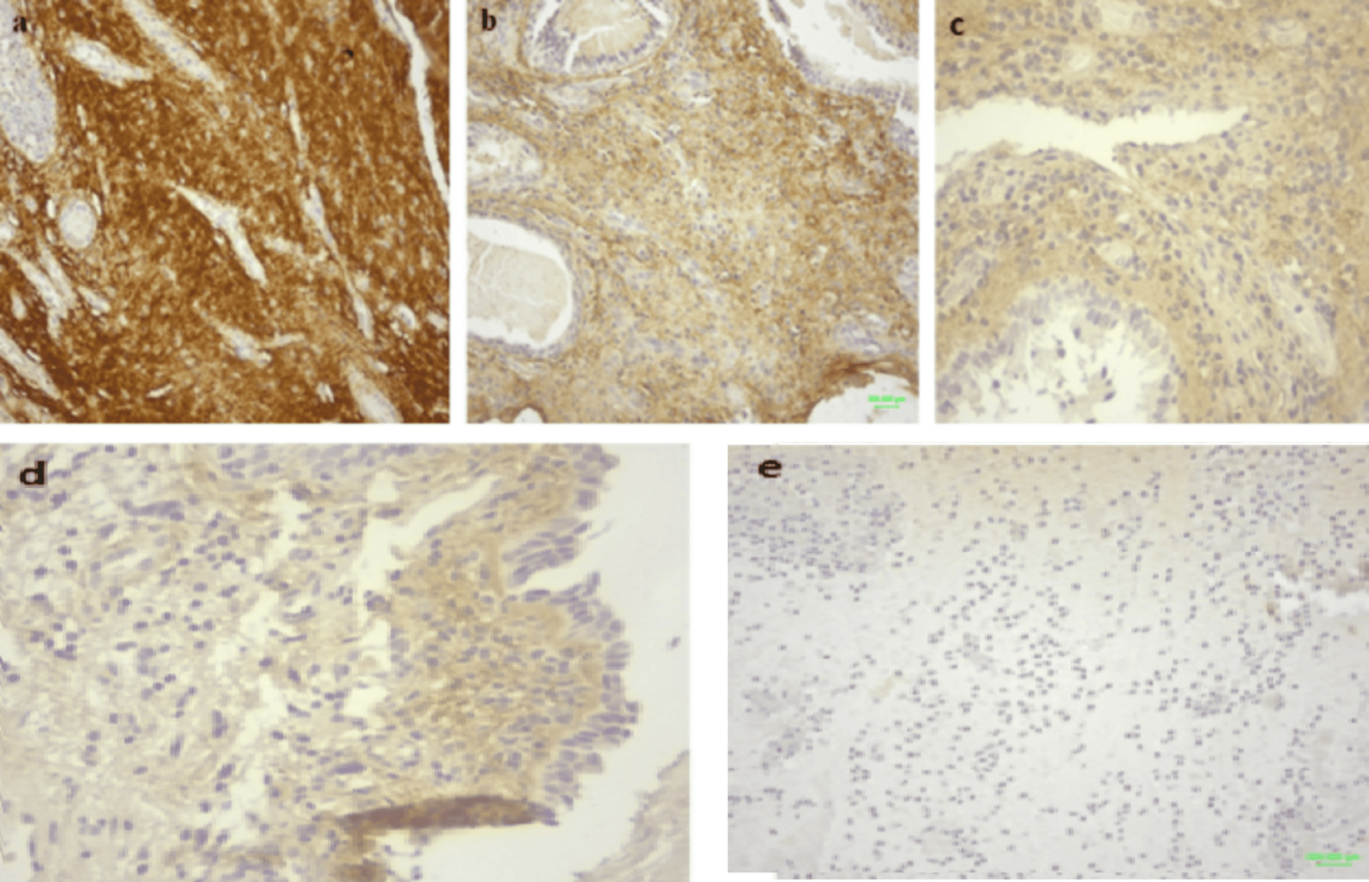
Immunohistochemistry of periostin in control (top row) and study group (bottom row). Periostin is expressed in the extracellular matrix, in the lamina propria and the thickened basement membrane in CRSwNP (magnification x20). (a) Intense staining in the control group. (b) Moderate staining in the control group. (c) Moderate staining in the study group. (d) Mild staining in the study group. (e) Absence of staining in the study group CRSwNP: chronic rhinosinusitis with nasal polyps

There is a statistically significant difference between the control group and the nasal polyps with preoperative administration of corticosteroids and antibiotics (study group) in terms of periostin expression (increased in the former) (p < 0.001) (Table 4). The group that received the treatment has a significantly lower score than the group that did not receive treatment (treatment group means H-score = 112.7 (SD = 40.4)/control group mean H-score = 159.4 (SD = 42.3)) (Figures 1, 2).

**Table 4:** Results from the comparison of H-score between the study and control groups CRSwNP: chronic rhinosinusitis with nasal polyps; SD: standard deviation; IQR: interquartile range

CRSwNP with preoperative corticosteroid administration (study group)		CRSwNP without preoperative corticosteroid administration (control group)		p value
Mean (SD)	Median (IQR)	Mean (SD)	Median (IQR)	
112.7 (40.4)	125 (80-140)	159.4 (42.3)	152.5 (125-185)	<0.001

Thus, nasal polyps with preoperative administration of systemic corticosteroids and antibiotics show reduced immunoexpression of the inflammatory marker periostin compared with those without any premedication.

Relationship Between Periostin Immunoexpression and Vascularity (Microvessel Density, Optical Density of Vascular Endothelial Marker) in CRSwNP With Preoperative Corticosteroids

The correlation of periostin, based on H-score, with MVD and optical density was also tested (Table 5), but no significant associations were detected (Rho = -0.02, p = 0.907; rho = 0.07, p = 0.713, respectively).

**Table 5:** Spearman’s correlation coefficients based on H-score with MVD and OD OD: optical density; MVD: microvascular density

Parameter	MVD	OD
Rho	-0.02	0.07
p value	0.907	0.713

## Discussion

Corticosteroids have anti-inflammatory and immunosuppressive properties. They prevent various cells, such as T-cells, eosinophils, and epithelial cells, from producing cytokines[4,5]. According to recent research [6-8], corticosteroids and antibiotics have a substantial impact on nasal symptoms, systemic markers of inflammation, and the growth of nasal polyps. Different pathogenetic inflammatory pathways are affected by each of the aforementioned parameters. Preoperative corticosteroids may lessen edema and inflammation and, as a result, reduce the growth of nasal polyps due to the aforementioned effects.

We studied, for the first time, the effect of immediate preoperative corticosteroid and antibiotic therapy on inflammation in CRSwNP using histological nasal polyp specimens. We evaluated the inflammatory biomarker periostin separately in histological tissue of nasal polyps from patients with preoperative administration of corticosteroids and antibiotics and from patients who didn’t receive any pretreatment.

Periostin (POSTN) is an innovative and recently studied biomarker. Studies involving periostin have increased rapidly over the past decades. It is an extracellular matrix protein that was first identified from an osteoblast cell line; IL-4 and IL-13 stimulate airway epithelial cells to produce it. It is an extracellular matrix protein that was first identified from an osteoblast cell line; IL-4 and IL-13 stimulate airway epithelial cells to produce it. Periostin contributes to the remodeling of the upper respiratory system [9]. It was initially identified for its significant function in myocardial remodeling and healing after myocardial infarction [9,10]. It also regulates fibrosis and collagen deposition. Periostin overproduction in the nasal mucosa is significantly expressed in chronic Th2 inflammation, including asthma [10,11], and has been linked to polyp formation [12].

According to Maxfield et al. [13], a significant correlation between high serum periostin levels and the existence of nasal polyps in patients with CRS has been established. Periostin may be a new target for upcoming therapeutic approaches and seems to be a novel molecular biomarker for the classification of CRS into at least two different molecular endotypes. Periostin has been linked to the pathophysiology of eosinophilic chronic rhinosinusitis (ECRS) and is a more valuable biomarker than eosinophils in ECRS, according to recent research by Sato et al. [14]. It was demonstrated to be a significant indicator for both postoperative recurrence of nasal polyps and pathological severity of ECRS.

Finally, in the present research study, we sought, for the first time, to determine whether there is a correlation between periostin immunoexpression and vascularity in CRSwNP patients who received preoperative corticosteroid administration.

According to recent literature, no study on CRSwNP has been conducted. Kula et al. [15] examined the potential effect of periostin on vascularity, thoroughly investigating its role in angiogenesis and inflammation in colorectal cancer. Based on the literature data, periostin expression is upregulated in poorly vascularized tissues undergoing hypoxia [16].

Further investigations into the association between periostin and cytokines are necessary to understand the interactions between tumors and immune responses in the tumor microenvironment, which could be useful for developing new, more targeted therapies.

However, steroids are known to increase the effects of endogenous noradrenaline and adrenaline, which consequently causes vasoconstriction. This leads to improved surgical field quality, decreased intraoperative hemorrhage, and decreased vascular permeability [17].

According to recent research, the administration of perioperative corticosteroids also shortens operating time, reduces blood loss, and enhances the quality of the surgical field [18-20]. Other drugs have not been demonstrated to impact on the surgical field or result. A poor surgical field, often caused by excessive bleeding or anatomical complexities, can lead to increased operative time, higher rates of intraoperative complications, and poorer long-term results. It is unknown whether systemic corticosteroids have an additional effect beyond nasal corticosteroids. The steering group for the European Position Paper on Rhinosinusitis and Nasal Polyps recommends using (nasal) corticosteroids prior to ESS [21]. The following scale is used to evaluate the intraoperative blood loss and the visual surgical field quality during ESS (Table 6) [22,23].

**Table 6:** Quality of the visual surgical field Source: Adapted with permission from the original publisher of [24]

Grade	Assessment
0	No bleeding (cadaveric conditions)
1	Slight bleeding, no suctioning required
2	Slight bleeding, occasional suctioning required
3	Slight bleeding, frequent suctioning required; bleeding threatens surgical field a few seconds after suction is removed
4	Moderate bleeding, frequent suctioning required and bleeding threatens surgical field directly after suction is removed
5	Severe bleeding, constant suctioning required; bleeding appears faster than can be removed by section; surgical field severely threatened and surgery usually not possible

Further larger cohort studies are required to validate this recommendation in histological preparation.

Limitations

The limitations of our study include the small sample size. The sample consisted of CRSwNP, that is, pathological inflammatory tissue. There was no access to a histological preparation of normal nasal mucosa, which is rarely removed even in other classic nasal surgeries (septoplasty/rhinoplasty, etc.). This would allow a comparative study of tissues from normal nasal mucosa and additional research. This is because all cases involved patients who underwent surgical treatment after failure of adequate conservative treatment by the ENT Clinic. The sample was selected randomly based on data completeness and the material's workability.

Moreover, there was no follow-up of all patients to further correlate the results with other clinical and laboratory parameters. Finally, the intraoperative bleeding and the quality of the visual surgical field during ESS are also significantly influenced by the severity, progression of the disease, and the history of reoperations due to recurrences.

## Conclusions

This study supports the possible anti-inflammatory role of the immediate preoperative corticosteroids and antibiotics in CRSwNP; consequently the reduction of the degree and inflammation intensity may impact on the surgical outcome during ESS (less blood loss, reduced overall surgical time and better quality of the visual field). Further cohort studies are required to validate this recommendation and to determine the optimal dosage and the duration of the pretreatment in patients with CRSNP. There are also known side effects from corticosteroids, and therefore each patient, should be considered as an individual case.
